# A pipeline for the systematic identification of non-redundant full-ORF cDNAs for polymorphic and evolutionary divergent genomes: Application to the ascidian *Ciona intestinalis*

**DOI:** 10.1016/j.ydbio.2015.05.014

**Published:** 2015-08-15

**Authors:** Michael J. Gilchrist, Daniel Sobral, Pierre Khoueiry, Fabrice Daian, Batiste Laporte, Ilya Patrushev, Jun Matsumoto, Ken Dewar, Kenneth E.M. Hastings, Yutaka Satou, Patrick Lemaire, Ute Rothbächer

**Affiliations:** a Institut de Biologie du Développement de Marseille Luminy (IBDML, UMR 6216), CNRS, Université de la Méditerranée, Parc Scientifique de Luminy, Case 907, F-13288 Marseille Cedex 9, France; b Gurdon Institute, Cambridge University, Cambridge, United Kingdom; c Montreal Neurological Institute and Departments of Neurology and Neurosurgery and Biology, McGill University, 3801 University Street, Montreal, Quebec, Canada H3A 2B4; d Department of Zoology, Graduate School of Science, Kyoto University, Sakyo, Kyoto 606-8502, Japan; e The Francis Crick Institute, Mill Hill Laboratory, The Ridgeway, Mill Hill, London NW7 1AA, UK

**Keywords:** Full-ORF, Functional genomics, Prediction pipeline, Ascidians, Transcriptomics, Human disease

## Abstract

Genome-wide resources, such as collections of cDNA clones encoding for complete proteins (full-ORF clones), are crucial tools for studying the evolution of gene function and genetic interactions. Non-model organisms, in particular marine organisms, provide a rich source of functional diversity. Marine organism genomes are, however, frequently highly polymorphic and encode proteins that diverge significantly from those of well-annotated model genomes. The construction of full-ORF clone collections from non-model organisms is hindered by the difficulty of predicting accurately the N-terminal ends of proteins, and distinguishing recent paralogs from highly polymorphic alleles. We report a computational strategy that overcomes these difficulties, and allows for accurate gene level clustering of transcript data followed by the automated identification of full-ORFs with correct 5′- and 3′-ends. It is robust to polymorphism, includes paralog calling and does not require evolutionary proximity to well annotated model organisms. We developed this pipeline for the ascidian *Ciona intestinalis*, a highly polymorphic member of the divergent sister group of the vertebrates, emerging as a powerful model organism to study chordate gene function, Gene Regulatory Networks and molecular mechanisms underlying human pathologies. Using this pipeline we have generated the first full-ORF collection for a highly polymorphic marine invertebrate. It contains 19,163 full-ORF cDNA clones covering 60% of *Ciona* coding genes, and full-ORF orthologs for approximately half of curated human disease-associated genes.

## Highlights

• Ascidians foster functional genomics by compact genomes and fixed cellular lineages.
• A resource of 19.000 GATEWAY full ORF clones was generated for *Ciona intestinalis*.
• Novel methods support automated finding of coding 5′ ends and paralog distinction.
• The strategy is robust to polymorphism and poorly annotated genomes.
• Half of human disease associated genes are covered by full ORF *Ciona* orthologs.

## Graphical abstract

**Figure:**
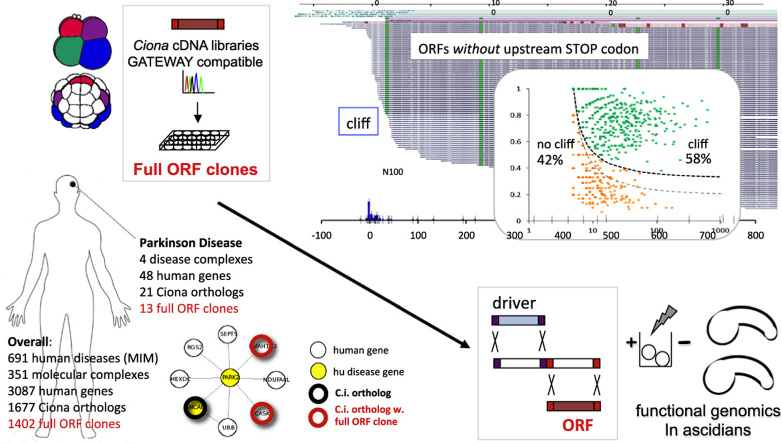

## 1 Introduction

Biomedical research has greatly benefited from the study of invertebrate model organisms. Modelling cellular networks in invertebrate model organisms with genomic resources, including collections of cloned open reading frames (or ORFeomes), led to an improved understanding of fundamental cellular processes and their malfunctioning (for review, see Vidal et al. (2011)). In parallel, the analysis of patterns of protein conservation over large evolutionary time scales can identify functionally relevant domains, although important domains can be lost in organisms as distantly related to vertebrates as the protostomes *Drosophila melanogaster* and *Caenorhabditis elegans*.

Marine environments are home to a rich diversity of animals covering all phyla, some of which have made major contributions to our understanding of biological processes (Cubitt et al., 1995; Doree and Hunt, 2002; Kandel, 2001) and their evolution (Garfield et al., 2012; Hinman and Davidson, 2007; Putnam et al., 2008; Simakov et al., 2013). Ascidians are marine invertebrate chordates that share a tadpole-like developmental stage with vertebrates, yet diverged long ago from the vertebrate lineage (Lemaire, 2011). Interestingly, this evolutionary conserved larval body plan is built from a much more compact genome than vertebrates, with small intergenic regions, compact genes (a few of them duplicated) and short *cis*-regulatory modules. As a consequence, the regulatory part of the genome, including transcription factor genes and the *cis*-regulatory sequences where they bind to orchestrate transcriptional networks, is particularly small. A likely adaptation to a compact genome is that around 20% of ascidian genes are organised into operons (Satou et al., 2008a). This phenomenon is associated with the extensive use of trans-splicing (Matsumoto et al., 2010), a mechanism whereby a unique short splice leader (SL) sequence is spliced onto the 5′ end of many mature mRNAs (Hastings, 2005; Satou et al., 2006).

*Ciona intestinalis* is currently the major ascidian model organism. The simple *Ciona* embryos can be efficiently manipulated, microinjected and electroporated in batch, which, combined with their genomic simplicity, makes them one of the most powerful chordate systems for functional genomics approaches. This has allowed a partial deciphering of the early Gene Regulatory Networks (Imai et al., 2006) and extensive characterisation of several hundred *cis*-regulatory sequences (Tassy et al., 2010). Recent studies suggest that, in addition to their role in helping us understand the fundamental processes of ascidian developmental biology, *C. intestinalis* may help shed light on the origins of vertebrate features (Abitua et al., 2012; Kaplan et al., 2015; Mazet et al., 2005). As expected from their phylogenetic vicinity to vertebrates, ascidian proteins may be active when expressed in vertebrate systems (Davis and Smith, 2002; Marcellini et al., 2003). Finally, ascidians are promising organisms to understand the molecular mechanisms underlying human pathologies (Virata and Zeller, 2010) and tissue regeneration (Jeffery, 2015; Rinkevich et al., 2013).

In order to streamline *in vivo* functional genomics approaches in *Ciona*, we have previously established, and successfully used, the GATEWAY cloning system for the functional analysis of coding and non-coding regions in this species (Lamy et al., 2006; Pasini et al., 2006; Rothbächer et al., 2007). This encouraged us to generate a set of GATEWAY expression vectors adapted for mRNA injections or electroporations in *Ciona* and other metazoans (Roure et al., 2007). This system is suitable for handling large numbers of clones in medium-throughput gain- or loss-of-function screens. The required companion for such an approach is as complete as possible a collection of full-ORF cDNAs. An initial collection of 13,364 unique cDNA clones, built from a large set of ESTs, has previously been released (Satou et al., 2002). This collection, however, includes a substantial fraction of incomplete cDNAs, and is constructed in a vector that is not compatible with the GATEWAY system.

**Fig. 1:**
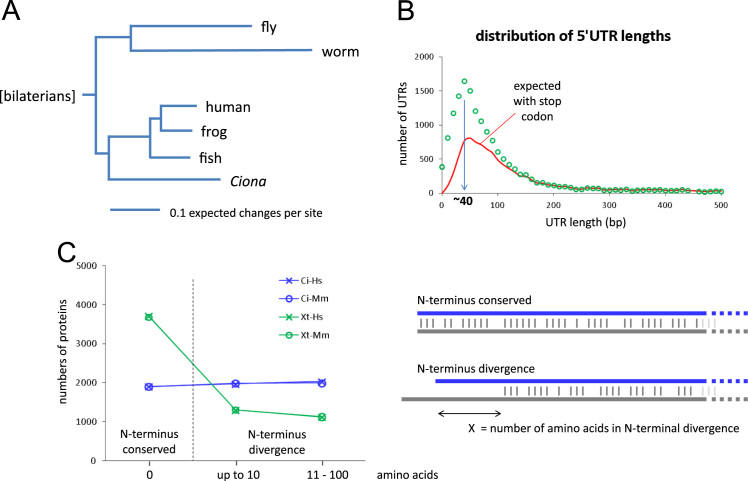
Coding genome of *Ciona intestinalis*. (A). Phylogenetic position of *Ciona intestinalis* relative to major model organisms, with branch length indicating degree of amino acid divergence (adapted from Putnam et al. (2007)). (B) Length distribution of 5′ UTRs in *Ciona intestinalis* determined from assembled EST sequence where open reading frame is probably complete. Red line indicates the proportion at any given length expected to include at least one in-frame stop codon. (C) Lack of conservation of N-terminus of *Ciona intestinalis* proteins relative to well annotated model systems, and compared to *Xenopus tropicalis*. Comparison of BLASTp alignment data using sets of mutual orthologs between *Ciona intestinalis*, *Xenopus tropicalis*, and either human or mouse. Schematic of BLAST alignments indicates how N-terminus divergence is measured.

In spite of significant scientific interest, there is to our knowledge no marine invertebrate species for which a systematic collection of full-ORF cDNA clones has been developed. A collection of 24,020 cDNA clones was generated in the cephalochordate *Branchiostomae floridae* (Yu et al., 2008), but no specific attempt was made to select only full-ORF clones, nor to distinguish between recent paralogs and highly polymorphic loci. This may in part be due to the challenge of marine invertebrate genomes: recognition of open reading frames is made harder by the large evolutionary distances to the available non-marine model organisms with substantially mature genome-scale protein annotation. In the present case, *C. intestinalis* diverged over 500 million years ago from the closest taxa with annotated genomes: vertebrates and cephalochordates (Putnam et al., 2008). Extensive protein divergence (Fig. 1A, adapted from Putnam et al. (2007)), contribute to the difficulty of identifying N-terminal coding sequences of many *Ciona* proteins by simple comparison to orthologous proteins in the well annotated vertebrate species (Fig. 1C), an issue worsened by typically short 5′ UTRs, often lacking upstream in-frame STOP codons (Fig. 1B). In addition, many marine invertebrates have high levels of polymorphism and undergo cryptic speciation: allelic variation in *C. intestinalis* within individuals can be over 1.5% (Dehal et al., 2002), and divergence between the two described subspecies can reach 12% in some loci (Caputi et al., 2007; Nydam and Harrison, 2010). This degree of variation significantly widens the range of sequence identity over which allelic variation at a single locus may be confused with sequence divergence between recent paralogs, and thus complicates gene referencing and non-redundant clone selection.

Overcoming these difficulties to generate a comprehensive and versatile full-ORF cDNA clone collection for *C. intestinalis* involved the construction and large-scale sequencing of novel GATEWAY compatible cDNA libraries but also, and most importantly, the development of specific algorithms, which this article describes. We started from the existing geneDistiller pipeline developed for vertebrates (Gilchrist et al., 2004), and extended it for application to highly divergent and heterozygous organisms. We have in particular developed a novel algorithm for automated 5′ end recognition and a method for distinguishing paralogous differences from allelic variation.

Here, we present these extensive conceptual improvements and characterize the ensuing first comprehensive collection of 19,163 full-ORF cDNA clone for a highly polymorphic marine invertebrate. The collection is organised into a set of fifty 384-well plates for copying and distribution; the identity and coordinates in the plate of the full-ORF clones can be found on each gene card page in the ANISEED database (www.aniseed.cnrs.fr), from which a table with the 384-well plate coordinates for each clone can be downloaded. This collection opens the way to functional genomics screens in ascidians and has been distributed to over 20 ascidian labs worldwide. Interestingly, we find that approximately 60% of confirmed human disease genes have orthologs in *C. intestinalis*, and we show that a large majority of human disease complexes are covered by at least one full-ORF clone in our collection.

## 2 Materials and methods

### 2.1 Construction of *cien* cDNA library

The *cien* cDNA library was generated from pooled mRNA taken from embryos at various stages of development from egg to neurula stage. Several animals were collected from the Northern Atlantic ocean near Roscoff (France), a region where populations of both type A and type B *C. intestinalis* subspecies are sympatric. The library was constructed using the Invitrogen GATEWAY system (CloneMiner™, Custom cDNA library Services, Invitrogen, cat. no. 11144-010), as follows. PolyA+ mRNA was extracted from pooled tissue samples, and cloned directly into the GATEWAY compatible pDONR222 vector using ATT linkers (Biotin-attB2-oligodT first strand primer), giving an unamplified, uncut large insert cDNA library, retaining 5′ and 3′ untranslated regions in addition to the coding sequences.. To assess quality and diversity we end-sequenced 3439 clones, and found 98% of sequence reads matched the genome in 2083 annotated gene loci. We found 5% of reads matched to gene loci not already covered by public ESTs. The library is referenced as NCBI/UniGene library ID 23002.

### 2.2 Assaying candidate cDNA libraries for diversity prior to deep sequencing

To complement the *cien* library, we investigated two similarly constructed GATEWAY libraries that were (a) from the Pacific *Ciona* population and (b) covering different stages/tissues from the *cien* library, and to which we had access for sequencing. These were the *cima8* (mature adult, NCBI/UniGene 15772, 37,489 public ESTs) and the *ciem8* (egg to larvae, NCBI/UniGene 15771, 35,518 public ESTs) from the laboratory of Yutaka Satou. Comparison with the first 100,000 sequences from the *cien* library, showed that the *cima8* ESTs matched more JGI v1.0 transcript models missed by the *cien* library sequences, compared to the *ciem8* library (by 1302–826), and was therefore likely to add more diversity to our collection.

### 2.3 Sanger sequencing of primary cDNA libraries

The GATEWAY cDNA clones were arrayed and sequenced by standard Sanger sequencing on equipment at JGI. All clones supplied were sequenced from both the 5′ and 3′ ends, with success rates passing the JGI submission quality threshold of 90% and 98% from the *cien* (also called XABT) and *cima8* (also called CBWU) libraries, respectively. The *cien* library, made from mixed early embryonic stages sampled from North Atlantic animals, was sequenced to a depth of 359,578 ESTs. The second, or *cima8* library, made from whole mature adults sampled from the Pacific population, was sequenced to a depth of 196,408 ESTs. Together these provided more than 500 million bases of long read sequence. EST sequences can be found in the EST section of GenBank.

### 2.4 Alignment of EST and cluster sequences to Kyoto Hoya (KH) *C. intestinalis* genome assembly and gene models

The current *C. intestinalis* genome assembly, called Kyoto Hoya (KH) (Satou et al., 2008b), was downloaded from the ANISEED database (http://www.aniseed.cnrs.fr/aniseed/download/download_data). The corresponding gene models, named KH gene models, were initially manually curated in 2008 (KH2008) and have have been successively refined in 2010 and 2012 (KH2010, KH2012 models). The KH2010 (http://ghost.zool.kyoto-u.ac.jp/datas/KH2010.1.KHGene.gff.zip) and KH2012 (http://ghost.zool.kyoto-u.ac.jp/datas/KH.KHGene.2012.gff3.zip) model sets used in this work are largely identical. The former was used for a more general quality assessment of the sequenced libraries, while the later was used for the more accurate analysis of clone coverage.

To estimate coverage of KH2010 gene models with ESTs, all available EST sequences were matched to the KH2010 transcripts model by best hit BLASTn analysis using an *e*-value threshold of *e*-20 and a word size of 20. EST sequences were then grouped by KH gene locus.

To identify putative exons, the full-ORF clone EST sequences were aligned to the KH assembly using the EST2genome model of the Exonerate alignment programme. We ran the search at low stringency to allow for the high level of sequence divergence between type A and type B strains, using the following parameter list: –model est2genome –gapopen-15 –bestn 1 –quality 85 –percent 33 –geneseed 200 –subopt false –hspfilter 100 –maxintron 10000.

### 2.5 Parallel de novo and reference EST clustering projects: (Fig. 2iii)

Clustering of ESTs was done largely as previously described (Gilchrist et al., 2004). The primary clustering project included all the available EST sequences. To minimise the effect of clustering errors on the identification of full-ORF clusters, we created three additional clustering projects each containing only those EST sequences mapping to one of three publicly available sets of computationally derived gene models at the time of clustering (Ensembl release 59, JGI v1.0, KyotoGrail2005). A fourth project assembled only those EST sequences which matched none of the gene models. These four additional clustering projects were analysed for overlooked full-ORF clones after analysis of the primary project clusters.

### 2.6 Dealing with heterozygosity and polymorphism: assembly stringency: (Fig. 2iv)

In order to handle the known high rate of polymorphism observed in *Ciona*, the primary clustering stringency threshold was reduced from 99% (Gilchrist et al., 2004) to 95% sequence identity. Under these conditions 1,074,949 sequences (generated in this project and retrieved from GenBank) that passed a basic quality threshold were assembled into 26,186 gene clusters and 9380 singleton ESTs.

### 2.7 Disentanglement of over-clustered back-to-back gene pairs: (Fig. 2v)

Analysis of the assembled EST clusters showed some erroneous co-clustering of pairs of opposite-strand, back-to-back genes with overlapping 3′ UTRs. These were detected after clustering, using protein BLASTx alignments to identify the respective coding regions, and EST strand orientation to detect the sense boundary for separation into pairs of clusters. For this, all assembled clusters were analysed for multiple open reading frames with significant matches to known proteins. Those with multiple open reading frames were further analysed for (a) a second downstream ORF with negative strand matches, and (b) the average orientation of assembled ESTs switching between predominantly positive strand to predominantly negative strand between the two ORFs (3′ ESTs were reverse complemented before assembly). Break points were identified in qualifying clusters at the strand switch position, and sequences mostly downstream of those points were transferred into new clusters with offsets and alignments inverted. New consensus sequences were generated for affected clusters. Using this approach we detected and split 476 pairs of genes.

### 2.8 Cliff algorithm for improved detection of clusters including 5′ end of mature messengers: (Fig. 2vi)

EST clusters containing the 5′ end of at least five clones are analysed for a concentration of 5′ ends suggesting the approximate start of transcription. A 100 bp sliding window, moved in 50 bp steps along the cluster, is used to determine the 100 bp interval with the highest number of 5′ EST ends in it: this peak interval starts at *x*, and contains $N_{100}$ EST ends. We then find $N_{1000}$, being the total number of 5′ EST ends in the 1 kb region 3′ of *x*. We consider clusters to potentially contain the start of transcription where $(N_{100}/N_{1000})\geq (1/3)+(2/3)\left(e^{-\;\log(Z-4)}\right)$, and *Z* is the number of 5′ EST ends in the cluster. That is, the fraction of 5′ end positions in the first 100 bp must be at or above a cluster size dependent limiting value, exponentially decaying according to log(cluster size), from 1.0 at size=5, to 0.333 for very large clusters (heavy dashed line in Fig. 3E). To measure the steepness of the cliff we use a fraction of the end positions in the peak interval, $N_{steep}$, where $(N_{steep}/N_{1000})\geq (1/5)+(4/5)\left(e^{-\;\log(Z-4)}\right)$, so that $N_{steep}\leq N_{100}\;$ (represented by the light dashed line in Fig. 3E). We then look for the steepest gradient, $m_{steep}$, over all sets of $N_{steep}$ consecutive 5′ end positions within the peak interval, where $m_{i}=\frac{N_{steep}}{∆x_{1}}$, and Δ*x_i_* is the distance along the cluster between the first and last positions of the *i*th set of consecutive positions. The position of the first of the consecutive points at the steepest position indicates the proximity of the start of transcription. We then generate two scores: $S_{peak}=\frac{N_{100}}{N_{1000}}$, and $S_{steep}=e^{-\{\frac{\ln(2)}{m_{steep}}\}}$, such that a ‘perfect’ cluster would score 1.0 on both scales. These are multiplied together and expressed as a percentage to give an overall cliff score=$S_{peak}\;\times S_{steep}\;\times 100$. Any cluster with an overall cliff score of 10 or more is treated as if there were an upstream stop codon verifying the 5′ end of the open reading frame.

### 2.9 ORF start detection: use of SL trans-splicing data (Fig. 2viii)

To take advantage of the presence of a unique splice leader (SL) sequence at the 5′ end of transcripts for ~50% of *Ciona* genes, we matched a set of 170,299 5′ 454 sequence reads starting from an SL sequence (Matsumoto et al., 2010) against our 5′ EST sequences using BLASTn. Clones containing the matched 5′ EST were present in 5049 top level clusters which were marked as full-ORF, and the clones made available to the clone selection process (Table 1).

### 2.10 ORF 3′end detection: (Fig. 2vii)

We found that genes with long mRNAs and/or low expression levels, frequently give rise to two clusters, one covering the 5′ end of the gene (upstream cluster), and the other the 3′ end (downstream cluster). To confirm the presence of the terminal stop codon of the open reading frame, or 3′ UTR sequence, in such downstream clusters, we devised two additional tests. First, we reduced the BLASTx *e*-value sensitivity threshold used in the rest of the project (0.001) to 0.1 to detect short stretches of coding sequence at the 5′ edge of (probably incomplete) contigs. Secondly, we identified stretches of well-assembled, 3′ UTR-like (no significant protein matches) sequence with stop codons in each frame, thus providing a definitive 3′ limit of any possible open reading frame. This region is then assumed to be downstream of the actual open reading frame. Similarly, contig sequences where no coding sequence is detected and the cluster is predominantly assembled (>66%) from 3′ ESTs, are assumed to be part of the 3′ UTR.

### 2.11 Alternative transcript picks: (Fig. 2x)

The geneDistiller pipeline (Gilchrist et al., 2004) allows gene clusters showing evidence of alternative splicing to be internally re-assembled with more stringent criteria into two or more transcript-based sub-clusters. These can then be used to facilitate transcript-based picks and enhance the functional coverage of the set. For genes with transcript-based sub-clusters, up to four clones were picked in total, with up to two clones per transcript, prioritising more abundant transcripts. This added 670 clones to the collection, representing 491 additional alternative transcripts over 449 genes.

### 2.12 Exon analysis for assessing redundancy: (Fig. 2xi)

The Exonerate mapping onto the current genome assembly of the EST sequences for all clones initially selected for the collection was used to identify likely consecutive exons in gene loci. Clones sharing the same set of putative exons were considered redundant; if there were more than two in any such redundant group the excess were removed from the list of clones selected.

### 2.13 Manual addition of clones for developmentally important genes

Visualisation of the automatically picked clones on a specific track of the ANISEED Gbrowse helped us to carry out some additional manual picking of clones, for genes of special interest to individual research projects, and whose full ORF was known from previous work. These genes were generally represented by only a few ESTs, and did not come through the automated pipeline well, but a tentative choice could be made after close visual inspection. 37 clones were manually added to the collection, including some important transcription factors and signalling molecules (ligands, co-factors, kinases and phosphatases).

### 2.14 Defining genes with highly conserved N-termini for RefSeq validation of cliff algorithm

To allow selection of genes with highly conserved N-terminal position, we used BLASTx (translated nucleotide query vs. protein database subjects) to match the set of downloaded *C. intestinalis* RefSeq transcripts to protein data from worm, fly, fish, chicken, mouse and human, using an *e*-value limit of 10^−10^ and taking the highest scoring match for each species. For each match we used the coordinates of the reported alignment to predict the likely start (P) of the coding sequence in the *Ciona* transcript from the other species protein. In the simple case where the protein aligns against the transcript from the start, P equals the position of the start of the alignment on the transcript. Where the reported alignment starts within the protein sequence, P is predicted by assuming that the length of the unaligned N-terminal part of the proteins is a conserved feature, and by calculating its position on the transcript sequence. Using standard BLAST alignment notation: P=query_start−3(subject_start−1), where query is the *Ciona* transcript and subject is the protein. Highly conserved proteins were then defined as those where proteins from all six other species gave the same value of P. There were 303 such transcripts in the *Ciona* NCBI RefSeq transcript set. Of these, 268 aligned to 227 EST contig sequences, which were both large enough (at least five 5′ ESTs) to apply the cliff algorithm and annotated by RefSeq as possibly full-ORF. These 268 RefSeq transcript sequences were used for our validation test.

### 2.15 Comparing EST contigs with RefSeq predictions for pipeline validation

For a wider validation of our full-ORF pipeline, we again used the *Ciona* RefSeq data, but this time compared the whole data set (excluding those annotated in GenBank as truncated) with the whole set of contig sequences for which full-ORF predictions were made. We combined BLAST alignments between our contig sequences and the predicted RefSeq coding sequences with assembly depth and other data to identify identical and differing ORF predictions.

To establish the relationship between our EST contigs and the *Ciona* RefSeq ORF predictions, we generated gapped and ungapped BLASTn alignments between these two data sets with an *e*-value limit of 10^−20^, using the coding sequence extracted from the RefSeq data with the given GenBank coordinates. Gapped alignments gave the best location of the start of the transcript sequence on the contig sequence, and ungapped alignments gave a more realistic per cent identity match. The analysed alignments contained 10,420 RefSeq transcripts and 10,035 EST contig sequences. From the alignment coordinate data, we first identified all those cases where the starts of the contig and RefSeq open reading frames were in agreement. 87% of the RefSeqs and 86% of the contigs agreed precisely on the predicted ORF. We then closely examined the remaining discrepant cases one at a time. For each case where we could identify the likely cause of the discrepancy we devised a logical test based on the available data to find similar cases in the unexamined remainder, and then continued with the next unexplained discrepancy. Where there were different predictions and we thought we could discriminate, we found 532 (5.1%) of the analysed RefSeq ORFs were likely to be incorrect, compared to 231 (2.2%) of the contigs. In some cases, especially where the RefSeq and contig sequences diverged, it was not obvious where the fault lay, and we marked up those on both sides as equally suspect. About 4% of ORF predictions fell in this category.

### 2.16 Sequence alignment for coverage of Ciona genes as a function of transcript length and relative abundance

Starting from the BLASTn alignment of all available ESTs to KH2010 transcript models, we estimated the relative expression level of each gene by summing up the numbers of ESTs from all GenBank deposited ESTs mapping to the transcripts in each gene model. To evaluate the relationship between abundance and coverage, we divided the 15,254 KH loci ranked by EST number into 20 equal bins. For transcript size estimation, we used the length of the longest predicted protein in each gene.

### 2.17 GO analysis of KH genes covered with a full-ORF clone

GO annotation of KH gene models was carried out as part of the ANISEED annotation pipeline (Tassy et al., 2010). Briefly, KH model proteins were analysed with InterproScan (Hunter et al., 2009; Zdobnov and Apweiler, 2001) for predicted domains. Using the curated InterPro2GO annotations (http://www.ebi.ac.uk/GOA/InterPro2GO, accessed 27.01.09.) we generated GO terms associated with the protein domains of each KH gene. Knowing which KH gene had associated full-ORF clones, we could compare the GO term distribution in full-ORF clone covered (or not covered) vs. all KH genes. To facilitate this analysis and the interpretation of its results, we first converted GO terms to GO Slim (http://www.geneontology.org/GO.slims.shtml; goslim_generic version 1.2, date 26.03.2008, accessed 2009) using map2slim (http://search.cpan.org/~cmungall/go-perl/scripts/map2slim). We also did a GO enrichment analysis comparing GOs of full-ORF clone KH against GOs for all KH genes. For this we did a hypergeometric test with Benjamini–Hochberg multiple testing *p*-Value correction using the BINGO software (Maere et al., 2005).

### 2.18 Finding *Ciona* orthologs of human disease genes

Human disease gene data were obtained from: http://www.cbs.dtu.dk/suppl/dgf/disease_complexes/index.php (Lage et al., 2008). A second set was obtained from the DisGeNET Database, GRIB/IMIM/UPF Integrative Biomedical Informatics Group, Barcelona (http://www.disgenet.org/). We restricted analysis of this database to the manually curated set (http://www.disgenet.org/ds/DisGeNET/results/curated_gene_disease_associations.tar.gz, retrieved April 2015) of 7108 human disease associated genes (Pinero et al., 2015). For 6012 genes, HGCN symbols (DisGeNET data) could be converted to ENSEMBL identifiers using data extracted from Biomart (ENSEMBL Release 79). Diseases associated with a complex (Data from Lage et al. (2008)) were transferred to the human genes belonging to that complex. Using Inparanoid (Remm et al., 2001), we identified orthologous relationships between the 14741 *Ciona* KH genes (53,203 peptides) and the 23,289 known protein-coding gene models in human (ENSEMBL v70, 104,785 peptides). These orthology relationships were the basis for the association between the subset of human disease gene models, *C. intestinalis* KH2012 models and their associated full-ORF clones.

## 3 Results

### 3.1 General overview of the procedure

The method follows, and uses large parts of, the geneDistiller pipeline previously developed for EST clustering and clone picking in *Xenopus tropicalis* (Gilchrist et al., 2004) and *Sus scrofa* (Gorodkin et al., 2007; Nygard et al., 2010). We will thus not repeatedly cite these publications in the following sections. Rather, we will focus on significant improvements, as well as specific adaptions for the *C. intestinalis* model system, that have advanced the method. The rationale and general approach for these modifications are described below, with detail provided in Section 2 where required.

As primary input for this project we generated a large and diverse set of end-sequenced cDNA clones, from which we identified and physically selected representative full-ORF clones for our collection. These were constructed with the Invitrogen GATEWAY system (Hartley et al., 2000; http://www.lifetechnologies.com) for optimal cloning flexibility (Section 2). A new mixed early embryonic stages GATEWAY-compatible cDNA library was constructed for this project from North East Atlantic animals, which was complemented by a pre-existing mature adult GATEWAY cDNA library from West Pacific animals. These two libraries were arrayed and Sanger sequenced from both ends to a very deep level. The resulting 500 million bases of long read sequence likely capture substantial sequence variation in the species, as the N.E. Atlantic library was made from both type A and type B *C. intestinalis* individuals, while the pacific library was solely made from type A animals. The resultant sequence information was passed to the adapted and enhanced geneDistiller pipeline to identify full-ORF clones.

**Fig. 2:**
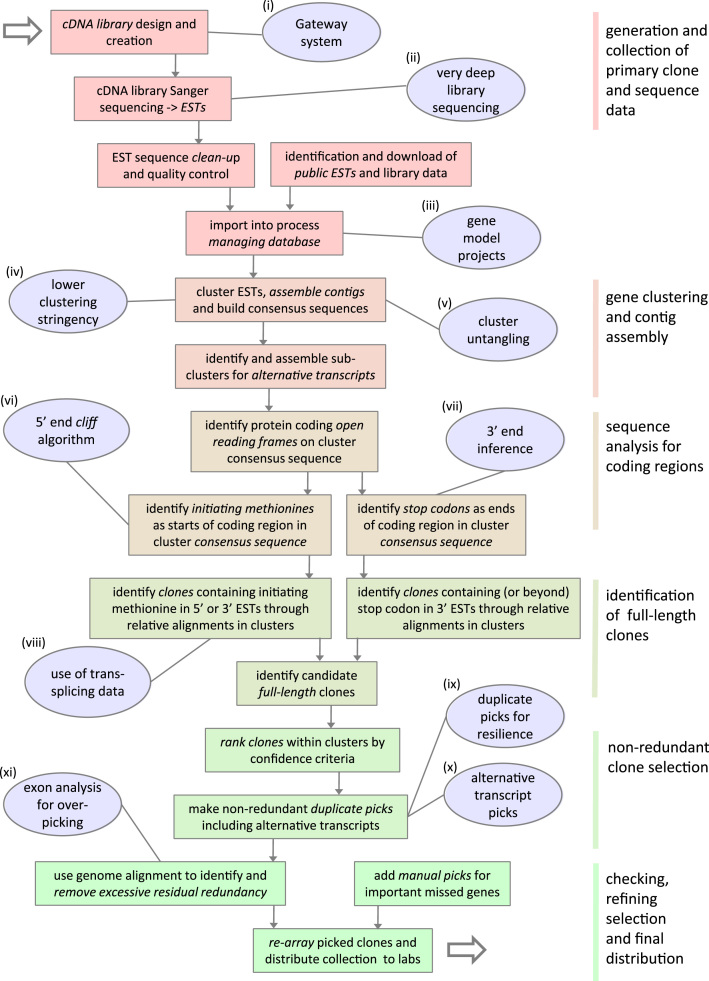
Workflow of full-ORF pipeline showing novelties. Boxes show schematic workflow of the geneDistiller pipeline for the analysis and definition of full-ORF clones from a large collection. Colour blocks show major sections of process. Ovals indicate important additions or updates added in this work, the two most important conceptual novelties (vi, xi) are described in the text. The other improvements are detailed in Section 2.

The general organisation of the workflow for defining and selecting full-ORF clones was as follows (Fig. 2). Sequences from our GATEWAY libraries (5′ and 3′ ESTs) were pooled with publicly available transcripts (ESTs), and computationally assembled into gene-based clusters, without reference to the available genome assembly. Computationally derived consensus sequences from these clusters were used to identify the starts and ends of the protein coding regions for each gene. The alignments of individual ESTs relative to the identified open reading frame (ORF) within each gene cluster identified candidates from amongst our GATEWAY clones likely to contain full ORFs. Two clones were provisionally selected per cluster/transcript. We included additional clones for alternative transcripts where found, and manually added a small number of clones for developmentally important genes with known ORFs, and known to be missed in the automated process.

### 3.2 Novel additions to the method

It has been noted that no single test successfully detects the majority of full-ORF clones, and that the best approach is to combine multiple tests (Strausberg et al., 2002). In this spirit, and to take the specifics of *C. intestinalis* into account, we added eight new analysis steps or tests to the vertebrate pipeline (Fig. 2iv–xi). The first class of new tests improved the assembly of accurate contigs from EST data, taking into account the high rate of polymorphism among *Ciona* individuals (iv), and the fact that because of increased gene density in *Ciona* compared to vertebrates, back-to-back genes on opposite strands may overlap in their 3′ UTR regions, leading to clustering errors (v). We next improved the identification within cluster sequences of the initiator methionine (vi), a task complicated by the shortness of *Ciona* 5′ UTRs and by extensive protein divergence, and of stop codons (vii). This information, combined with trans-splicing information (viii), was used to select candidate full-ORF clones. From these clones, we selected two clones for each gene to reduce the chance of gene loss over time in the collection (ix), and, where possible, picked clones corresponding to alternative transcripts. Finally, we refined the clone list by correcting for over-picking in the case of highly polymorphic genes: picked clones were mapped to the genome assembly, allowing us to verify that clones predicted to correspond to distinct genes indeed mapped to different loci (xi). Table 1 shows the relative contribution of each extension to our ability to confidently identify full-ORF clones.

Each of these improvements is detailed in Section 2, and the following sections will focus on solutions for the automated identification of the 5′ end of ORFs and on the distinction of paralogs, as these approaches had the largest impact on the quality of the generated clone collection, and may be conceptually applicable to other model organisms.

### 3.3 ORF start detection: the novel ‘cliff’ algorithm

**Fig. 3:**
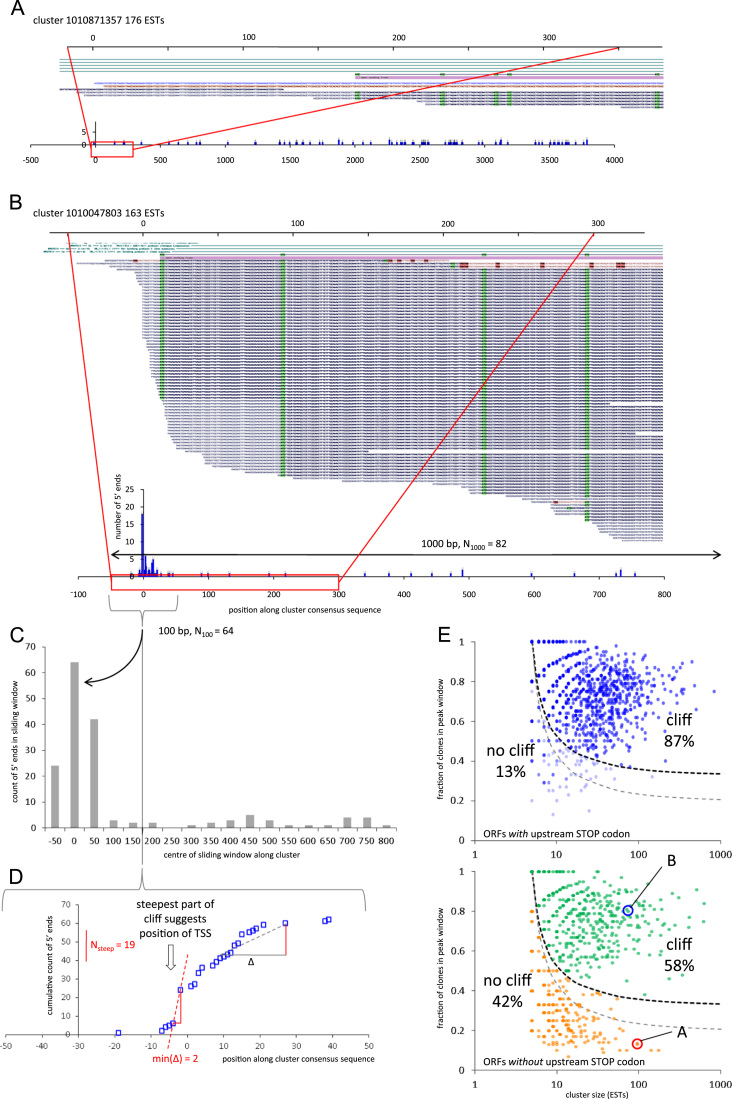
‘Cliff’ algorithm for confirming full-ORF status. A concentration of the positions of 5′ ends of clones in assembled clusters identifies the likely start of transcription, which is, by definition, upstream of the start of translation. (A) Cluster with 176 ESTs showing truncated open reading frame and no start of transcription. (B) Cluster with 163 ESTs showing ‘cliff’ of 5′ end positions likely containing the start of transcription. (C) Cliff finding: histogram of numbers of 5′ ends in sliding windows of 100 bp determined every 50 bp along 1000 bp of cluster, and used to find the ‘peak’ region of 5′ end density (*N*_100/_*N*_1000_). (D) Cliff steepness and transcription start site (TSS) prediction: analysis of cumulative 5′ end count across ‘peak’ 100 bp window, used to find the steepest part of the cliff for a determined fraction of reads in the window. (E) Cliff threshold: plots to test the cluster size dependent term for the limiting value *N*_100/_*N*_1000_, used to determine the presence of a ‘cliff’ and hence the likely start of translation (see text). The heavy dashed line follows the form $\frac{1}{3}+\frac{2}{3}\left(e^{-\;\log\left(Z-4\right)}\right)$ where *Z* is the clusters size (number of ESTs). Individual EST clusters (spots) are plotted according to their ‘peak’ of 5′ ends (*N*_100/_*N*_1000_) on the *y*-axis, and cluster size (*Z*) on the *x*-axis; those falling right and above of the limiting curve are assumed likely to contain sufficient cliff and the start of transcription. (Upper panel) Verification of cliff algorithm: (blue dots) clusters with upstream stop codon confirming open reading frame, showing score is a good predictor of full-ORF status. (Lower panel) Clone selection with cliff algorithm: clusters without upstream stop codon, showing clear bimodal distribution with cluster consensus sequences assumed full-ORF (green) and those assumed truncated (orange). Spots corresponding to the example genes in panels A and B are marked. The light dashed line shows the curve used to determine the proportion of 5′ ends in the peak window used to look for the steepest section of the cliff (see D).

Following EST clustering, clusters with plausible open reading frames may be found, which on inspection can be shown to be truncated, usually at their 5′ end (Fig. 3A). The identification of true starts of translation is sometimes facilitated by the chance presence of an upstream, in-frame stop codon. However, these are often absent in the short 5′ UTRs of the compact *Ciona* genome (Fig. 1B): the most common length of 5′ UTR sequence is ~40 bp (Fig. 1B), where the chance of not finding a stop codon in a given frame is close to 50%, decreasing to 1% at around 100 bp. To circumvent the issue of short UTRs, we added to our pipeline an alternative strategy to detect clones with a near complete 5′ end, using a novel *cliff* algorithm based on our understanding of the likely behaviour of reverse transcriptase during cDNA library production (Fig. 2vi).

Although reverse transcription can go no further than the 5′ end of the mRNA, it may terminate randomly before that. Widely spaced 5′ EST starts at the 5′ end of an assembled cluster therefore suggest random termination of reverse transcription, likely incompleteness of the assembled open reading frame, and hence likely truncation of the clones making up the cluster. Conversely, the presence of a spatially concentrated group of 5′ ESTs extending to similar start positions suggests that the cluster is likely to contain the start of transcription, and this will manifest itself as a rapid drop-off, or cliff, in the aligned sequences (Fig. 3B).

The heuristic model we developed to apply this observation (mathematical formulations are presented in Section 2) proposes that if we look at the distribution of positions of the 5′ end of clones over the first 1000 bp downstream of the start of transcription, we should find a significantly higher concentration of 5′ ends within the first 100 bp (Fig. 3C). We chose a lower limit, for large clusters, that at least one third of the 5′ ends in the first 1000 bp be within the 100 bp region; with the required fraction increasing progressively to all 5′ ends for the smallest clusters tested (those containing the five 5′ ends of just five clones). Clusters with at least this fraction of 5′ ends in the first 100 bp would be considered likely to contain the start of transcription. A second calculation locates the start of the steepest part of the cliff within the first 100 bp, as an indication of the position of the likely start of transcription. These calculations are combined to yield a cliff score between 0 and 100 (Section 2 and Fig. 3D). Here we used an arbitrary and slightly conservative minimum threshold score of 10 to assign 5′ end complete status to 3687 clusters without an upstream stop codon. This test has the advantage of being independent of genome-based gene modelling, similar to the HKSCAN test introduced by MGC (Strausberg et al., 2002). Unlike HKSCAN, it is also independent of the actual length of the 5′ UTR sequence.

### 3.4 Validation of cliff algorithm and ORF predictions

We validated the cliff algorithm by making an internal comparison between open reading frames defined by upstream stop codons and those without, and by analysis against NCBI RefSeq (Pruitt et al., 2014) data using a set of 303 proteins with highly conserved N-termini across metazoa. In addition we validated the net output of the pipeline as a whole against the complete *Ciona* RefSeq data set.

To assess the usefulness of the cliff algorithm in defining the 5′ ends of transcripts we compared the distribution of the peak enrichment of 5′ ends (*N*_100_/*N*_1000_) with clusters size, comparing clusters with an in-frame stop codon upstream of the first ATG (which are therefore likely to be full-ORF) to those without a stop codon (which may be full-ORF or may be incomplete). These distributions are clearly different (Fig. 3E). In the stop codon limited case, 87% of open reading frames (above the cluster size threshold) fall in the region where they qualify for a non-zero cliff score. This validates the score as a useful marker of full-ORF status. In the case of the ‘open’ ORFs, we find that 58% fall within this region; the majority of these will therefore likely be full-ORF. There is a clear gap between the two groups in the ‘open’ ORF distribution at larger cluster size, supporting the suggestion that these groups are essentially different.

We have shown that cliff detection improves full-ORF detection within our own data set; to validate the cliff algorithm against other data we chose the NCBI RefSeq (Pruitt et al., 2014; http://www.ncbi.nlm.nih.gov/refseq) data set for *C. intestinalis*. This non-redundant, well-annotated set of transcript sequences, with on-going curation by NCBI staff, contains annotation of predicted coding sequence regions. Coding sequence coordinates are included in the GenBank data, and are additionally annotated where they are known to be truncated, at either the 5′ or 3′ end.

We first compared 268 un-truncated RefSeq transcripts that had a conserved start position in *C. elegans*, *D. melanogaster*, zebrafish, chick, human and mouse and corresponded to *Ciona* clusters expressed at a high enough level to apply our Cliff algorithm (227 clusters, see Section 2 for details). Eleven of these clusters showed no cliff (false negative), though they included the 5′ end of the coding sequence. Two clusters had cliffs predicted at an appropriate position upstream of the start of translation, but our predictions for the ORF start disagreed with the RefSeq data. In one case RefSeq is correct, and in the other, both predictions are likely incorrect. We found no examples of misplaced cliffs. In all cases where a cliff was determined it appeared to be a good indicator of the start of transcription. From this analysis, we confirm that the cliff score is a sensitive and accurate method for predicting the 5′ ends of transcripts, given sufficient EST abundance in the cluster. The false negative rate (full-ORF containing clusters with no cliff detected) is quite low at ~5%, whilst the false positive rate (clusters with annotated cliffs but not full-ORF) is very low (none detected in 227 analysed). In all cases analysed, the cliff was found at or upstream of the start of the open reading frame; the average distance being 64 bp, which corresponds well to the global estimate of 5′ UTR length detailed above. This also suggests that instances of blocking of reverse transcriptase by secondary structure of the mRNA, which could have limited the pertinence of our method by creating internal cliffs within the coding part of the cDNA, are rare events. The data described here includes cliff scores down to the minimum value of 1; for the actual clone picking we took a more cautious approach, using a threshold of 10.

We next tested how the ORFs defined in our clusters compared to all *C. intestinalis* RefSeq models. Using BLASTn we found alignments for 10,420 RefSeq transcripts against 10,035 EST cluster contig sequences, and, of these, 87% of the RefSeqs and 86% of the contigs agreed precisely on the predicted ORF. Where there were different predictions and we thought we could discriminate, we found 532 (5.1%) of the analysed RefSeq ORFs were likely to be incorrect, compared to 231 (2.2%) of the contigs. About 4% of ORF predictions diverged in a manner where it was difficult to assign an error either way, and in 1.8% of cases both were probably incorrect.

### 3.5 Reducing redundancy and resolving paralogs: the ‘exon method’

In analysing sequence data from mixed strain libraries, it can be difficult to discriminate between strain variants and paralogous genes (Gidskehaug et al., 2011; Kapustin et al., 2008; Vinson et al., 2005). Whilst clustering at reduced stringency (see Section 2) resolved the intrinsically high within-strain polymorphism, we still generally found two distinct clusters about 90% similar, matching the same locus, leading to over-picking at these loci. The smaller cluster in each pair was generally composed exclusively of sequences from the Atlantic population, and the larger one contained transcripts from both Atlantic and Pacific populations. This is consistent with the presence of sympatric populations of type A and B *C. intestinalis* individuals in the Roscoff area where the Atlantic specimen were collected (Caputi et al., 2007), and suggests that the small divergent clusters represent type B sequences. Knowing the source library of each clone in our EST clusters, we identified 2006 type B clusters containing predominantly (at least 90%) Atlantic sequences (5 or more ESTs), matching a larger cluster containing a more mixed source of sequences, with a sequence identity match of between 85% and 95%. The sequence alignment included at least part of the open reading frames of both clusters. The smaller cluster was on average ~1/4 the size of the larger cluster.

To resolve this problem, and to identify clones with different exon usage, we generated genome alignments for the EST sequences of picked clones from which we extracted likely exon locations for each clone (Fig. 2xi and Section 2). Sequences from paralogous genes mapped to different loci, whereas unnecessary duplicates, which mapped to the same locus but probably corresponded to divergent type A and type B sequences, were removed. In this way we removed around 5000 redundant picks, or 20% of the total clones preselected for picking.

### 3.6 Gene representation and coverage of the full-ORF collection

The utility of our full-ORF GATEWAY clone collection is determined to a large extent by its coverage of *C. intestinalis* protein coding genes. The previous sections indicated that full-ORF cluster sequences covered more than 10,000 RefSeq genes. As the ascidian community mostly makes use of the *C. intestinalis* Kyoto Hoya (KH) genome assembly and associated protein coding genes, refered to as KH gene models, released shortly after clone picking was completed (Satou et al., 2008a), we analysed the coverage of this gene model set by full-ORF clones. The KH assembly and KH gene models made use of all available sequence data for this species, including the EST data generated for this project. The KH gene models are therefore not entirely independent of the clone sequences we were analysing, although our full-ORF predictions were not used in the gene modelling.

**Fig. 4:**
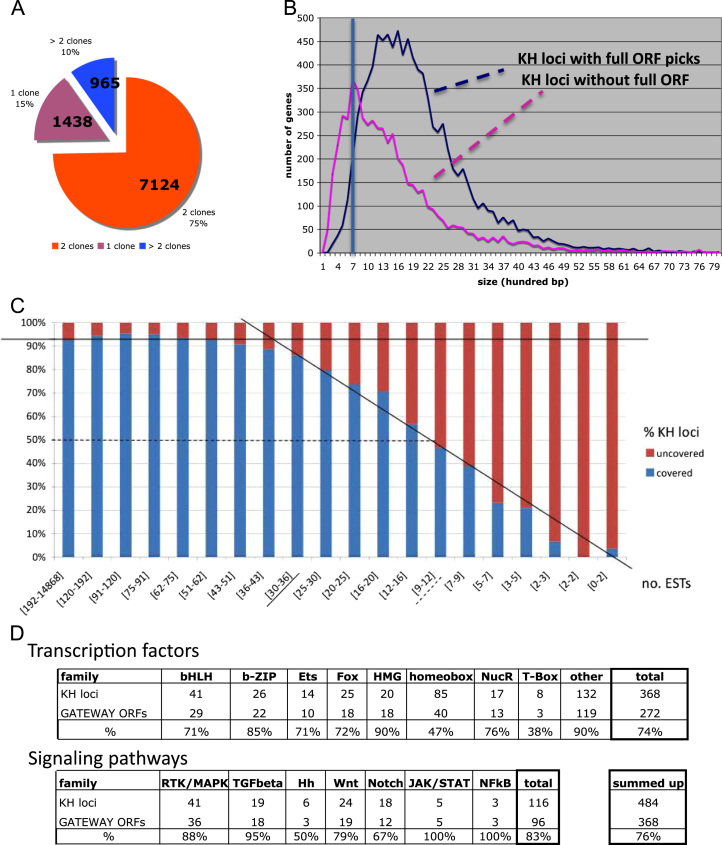
Full-ORF clone coverage of KH gene loci**.** (A) Proportion of KH loci covered by one or more full-ORF clones. (B) Size distribution of KH2010 loci covered or not by full-ORF clones. (C) Coverage relative to transcript abundance (EST count from all *C. intestinalis* cDNA libraries). (D) Full-ORF coverage of regulatory developmental genes.

We found that 99.3% (18,978/19,107) of our picked clones mapped at least one EST to the KH genome assembly. 97.3% of the clones (18,596/19,107) mapped to the locus of one of the 15,273 KH2012 gene models, covering 59.5% (9083/15,273) of these gene models. 75% of the covered genes were represented by two clones, 10% by more than 2 clones, and 15% by a single clone (Fig. 4A). In addition, there were 382 clones, which mapped to the genome assembly, but were not associated with a KH coding gene model; and 129 clones that did not map at all to the genome assembly. Assuming the coverage is unbiased, this suggests that there are at least ~500 *Ciona* coding genes (~2.5%) that are not captured in the KH gene model set, but for which we have a full-ORF cDNA clone in our collection.

To explore the reasons for not finding full-ORF clones for all genes, we first analysed gene coverage as a function of gene length. We found KH transcript models up to 8 kb in length with an associated full-ORF clone. For genes with transcripts longer than 1.5 kb we found the likelihood of coverage consistent with the overall rate of ~60%, whereas for shorter transcripts coverage decreased markedly (Fig. 4B). This may be partly due to a minimum 0.7 kb size selection in library preparation. It may however also point to artefacts in short KH models, in particular to the chance occurrence among non-coding sequences of small open reading frames that were incorrectly annotated as coding during the KH gene modelling process (Supplementary Fig. 1C). If we assume that the coverage remains constant for genuine smaller genes, this suggests that around 500 of the smaller KH gene models may not be genuine protein coding genes and may include UTRs of coding genes, or other genetic elements such as non-coding genes or transcribed enhancers (Marques et al., 2013).

We next explored the impact of the level of gene expression on coverage of KH models, as measured by the numbers of ESTs from all sources, mapping to each locus. We found that above 43 ESTs per locus (about one third of loci), 90% of KH genes are covered by the full-ORF collection. Below that, coverage declines as a linear function of the total number of ESTs matching the gene. Only 50% of KH genes matching 12 ESTs are covered and less than 10% of genes matching 3 ESTs (Fig. 4C). Our full-ORF clone set will therefore be somewhat, but not strongly, biased against genes represented at low abundance in the input libraries.

To explore the distribution of genes covered by our GATEWAY clone collection over major functional classes, we performed a GO analysis on covered KH gene models relative to all models (Section 2). We found a small number of both enriched and depleted GO Slim categories (v1.2 2008, Ashburner et al., 2000) (Table 2), although to a first approximation we expected our coverage to be unbiased. We know (above) that our coverage is in fact biased against both low abundance and very long genes, and it is plausible that certain general categories of genes may (particularly) be typically of low or high abundance. Bias may also have been introduced through the choice of cDNA library tissue, as they are solely derived from early development stages and mature adults. In addition, we note that there are more transcription factors (74%) and signalling molecules (83%) in the clone collection than we would expect by chance (60%) (Fig. 4D), indicating that the bias against low expression genes is sufficiently mild not to interfere with the identification of full-ORF clones for regulatory genes. There is no obvious reason why this should be so, although a small number of known missed genes in these categories were added in manually (see Section 2). Alternatively, it suggests that these genes may be more active in early development (Schep and Adryan, 2013). We conclude that there may be some bias in certain categories of genes, but that overall the full-ORF clone set is broadly representative of *Ciona* coding genes.

### 3.7 Representation of human disease genes

**Fig. 5:**
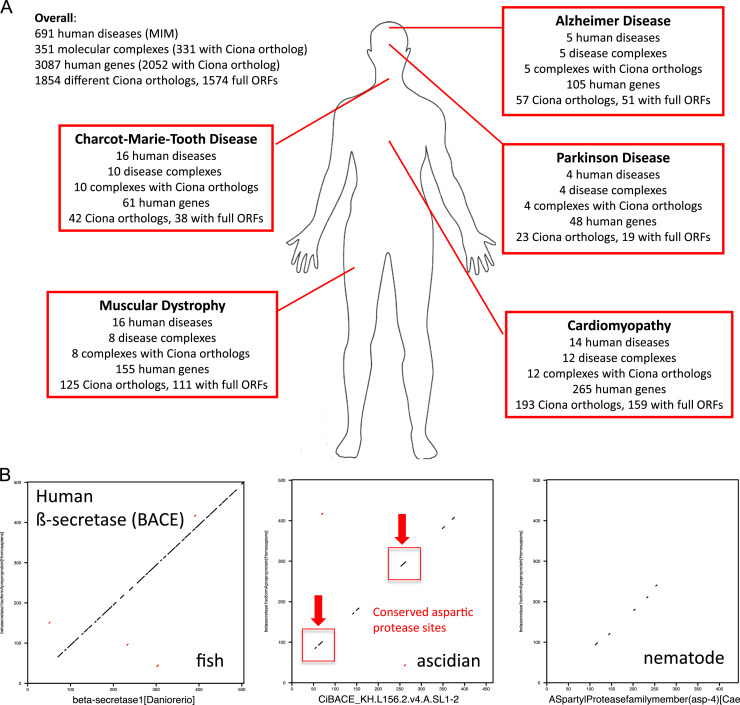
*Ciona* disease orthologs. (A) Human disease associated genes represented by *Ciona intestinalis* orthologs and full-ORF clones. Numbers of *Ciona* orthologs and full-ORF clones are depicted for five human diseases affecting neural or muscular tissue. Disease associated genes and disease complexes are from an integrated interactome (Lage et al., 2008) and contain potentially conserved functional modules to be analysed in simpler *Ciona* embryos. (B) Conservation of functionally relevant domains in *Ciona* despite little overall sequence conservation. Dotpath (EMBOSS) of human BACE-1 (GI:6912266) to orthologous protein sequences of zebrafish (GI:45387815), *Ciona* (KH.L156.2.v4.A.SL1-2) and nematode (GI:17549909).

Ascidians have been proposed as a model for human disease (Virata and Zeller, 2010). This is, in part, based on their intermediate evolutionary distance between human and the more tractable but phylogenetically distant model system of the fly and the worm. One consequence of this is that comparison of protein sequences between human and *Ciona* can be highly informative with conservation highlighting specific functional residues (Fig. 5B).

Increasing attention has recently been given to the predictive power of molecular interactions in integrated disease networks to suggest new disease genes and functional links. Notably, cellular components forming functional modules are hypothesis-building tools for particular disease phenotypes (see for review Barabasi et al. (2011) and Vidal et al. (2011)). In a similar way, human genes associated with similar pathologies and disease status, possibly co-expressed in similar tissues, have been clustered into putative functional modules of disease complexes (Lage et al., 2008), a strategy proven successful to discover novel links in human pathology (reviewed in Lage (2014)). Diseases, for which the whole complex is present in the *Ciona* genomes, and in our full-ORF collection, are thus promising for the development of an ascidian model. We thus characterized the repertoire of *Ciona* genes orthologous to human disease genes and their complexes as described by Lage and colleagues and in the independent human disease-associated gene database DisGeNET (Pinero et al., 2015)

To do this, we first established the scale of detectable orthology between these species. We found that 52% (7615) of *Ciona* KH genes have a human ortholog; corresponding to the 52% of *Ciona* genes found to have a zebrafish ortholog (Sobral et al., 2009). In the reverse direction, we found that 48% (10692) of human genes have a *Ciona* ortholog. From this we were able to identify 2052 (1854 unique) *Ciona* orthologs in a collection of 3087 human genes associated with disease in the high confidence, protein interactome of Lage and colleagues (Lage et al., 2008), and 3498 (3233 unique) *Ciona* orthologs in the more recent DisGeNET curated collection (Pinero et al., 2015) comparing 6012 human disease-associated genes. The slightly higher ortholog coverage (67% and 58%) compared to all genes (48%) is suggestive of the role that highly conserved genes may play in pathological developmental and homeostatic processes. Interestingly, in 63 out of the 351 non-redundant disease-associated complexes, all the genes in the complex have a *Ciona* ortholog. Our GATEWAY collection contains full-ORF clones for 1574 (85%) of the Lage disease-associated *Ciona* orthologs (Supplementary Table 1) and 2484 (79%) of the DisGeNET (Supplementary Table 2).

1745 human genes are in common (57% and 29%, respectively) between the two studies (labelled yes/Y versus no/N in Supplementary Table 2) of which 1170 (67%) have *Ciona* orthologs and 1011 (58%) full-ORF clones. In summary, our GATEWAY *Ciona* full-ORF clone collection covers human disease associated genes to 59% and 48% respectively, in the two datasets examined (with 33% and 17% that overlap).

Analysis of embryonic expression patterns of orthologous gene pairs has shown that these are most similar between these species in the developing muscle tissues and nervous system (Sobral et al., 2009), and suggests that diseases of these organs in particular may be usefully modelled in *Ciona*. We illustrated this (Fig. 5) for the following (Lage study) associated pathologies: Cardiomyopathy, Muscular Dystrophy, Parkinson's, Charcot–Marie–Tooth and Alzheimer's disease.

## 4 Discussion

We have described a transcript sequence clustering and full-ORF cDNA clone identification pipeline that can be applied to animals distantly related to the major model organisms, and with high intra-specific polymorphism, using *C. intestinalis* as a paradigm. The resulting collection of 19,107 clones covers around 60% of existing KH2012 gene models, with an acceptably small bias in terms of cDNA length, gene expression level and GO terms. 85% of the covered genes are represented by at least 2 clones. Importantly, we find that our fully automated ORF detection pipeline makes predictions of a quality at least equal to that of the curated RefSeq consortium. We further show that our full-ORF clone set extends the manually curated KH2012 gene model set by at least 500 genes. In practice, imperfections in the KH coding gene modelling process likely lead to an overestimate of modelled coding genes (see Section 2 and Supplementary Fig. 1C), suggesting that our GATEWAY clone set may cover more than 60% of true protein coding genes in this species. High interest genes such as transcription factors, and orthologs of Human disease genes are significantly better covered (74% and 84%, respectively).

While preliminary Gene Regulatory Networks have been reconstructed in *Ciona* by a loss-of-function approach, these networks only cover early development (Imai et al., 2006, 2009) and remain incomplete. The availability of the full-ORF collection described here, of a set of GATEWAY-compatible electroporation vectors (Roure et al., 2007) and of several hundreds *C. intestinalis cis*-regulatory sequences that can be used as drivers (Tassy et al., 2010), opens the way to expression cloning in *C. intestinalis*, a procedure that has led to the identification of master regulators of development in other systems (Chambers et al., 2003; Lemaire et al., 1995; Smith and Harland, 1992). Preliminary work (U.R and P.L, unpublished) indeed indicates that co-electroporation under the control of an early ectodermal driver of a single full-ORF *FGF9/16/20* (KH.C2.125) cDNA clone within a pool of 100 equimolar clones is sufficient to detect the early neural-inducing activity of this secreted factor in animal cells (Bertrand et al., 2003). The collection will also facilitate the use in Western European laboratories of *C. intestinalis* type B animals, a divergent sub-species (Caputi et al., 2007), whose genome locally differs by up to 12% from the published *C. intestinalis* sequenced genome (Nydam and Harrison, 2010), and which is prevalent in the North East Atlantic. The presence of full-ORF consensus cluster sequences from *C. intestinalis* type B animals, will in particular help design morpholinos (Satou et al., 2001) and CRISPR/Cas9 guide RNAs (Stolfi et al., 2014) for the corresponding genes. Finally, because the EST cluster assembly process does not rely on the KH genomic assembly or KH models, it provides an independent assessment method for their quality. Supplementary Fig. 1 provides four examples of the classes of residual assembly or modelling artifacts that the collection could help resolve. Finally, we have shown that, in spite of half a billion years of evolutionary divergence, around 60% of human disease-associated genes and their protein complexes have been conserved in *Ciona*, and most of these are represented in our full-ORF clone collection. The simplicity of ascidian embryos and the power of *Ciona* functional genomic tools can now be harnessed to shed light on the biochemical and cellular function of these medically important genes.

The usefulness of the algorithms described here could also extend beyond the *Ciona* community. In particular, our novel cliff algorithm for the identification of cDNA clones with a full-ORF 5′ end is sensitive, has a low false discovery rate, and should be particularly useful for organisms with short average 5′ UTRs. Consistently, we found that the cliff algorithm also provided a reliable assessment of the 5′ end of transcripts in *X. tropicalis* (Supplementary Fig. 2). Our approach is based on the clustering of long EST sequences generated by Sanger sequencing. This technology has now been superseded by the more cost-effective massive parallel short read sequencing (RNA-seq), which unfortunately does not give access to physical clones that can be organised into full-ORF collections. The exon detection method described in this article should be readily applicable to clusters assembled from short reads. Adaptation of the cliff algorithm may be more problematic as cDNAs are fractionated into fragments of a few hundred nucleotides prior to sequencing, and this may interfere with the detection of the cliff.

We note, however, that the assembly of short RNA-seq sequences into high quality full-ORF transcript predictions remains problematic (Steijger et al., 2013). In particular, the small length of sequenced cDNA fragments restricts the detection of multiple alternative exon usage, which can lead to a combinatorial increase in the number of putative transcript isoforms, and to a lack of clarity over which transcripts are real and/or most abundant. In addition, current assembly methods of short read RNA-seq data, such as Trinity (Grabherr et al., 2011), use a k-mer approach and do not generally provide depth/abundance information in their output: assembly depth has no clear meaning in this context, as highly abundant transcripts reduce to similar numbers of k-mers as much less abundant ones. We have however found in this study that assembly depth is very useful for assessing likely transcript 5′ ends (the cliff algorithm), as well as the relative abundance of different isoforms and the solidity of the contig assembly at key points. This problem has been recognised, and, for example, the Corset pipeline (Davidson and Oshlack, 2014) maps short reads back to contigs after assembly to assess expression levels. As a consequence of such limitations the NCBI UniGene database (Sayers et al., 2012) still exclusively defines representative gene transcripts on the basis of EST sequence clustering. Hybrid sequencing strategies, in which single-molecule sequencing of cDNA fragments of several kbs is corrected using short read RNA-seq are being developed to alleviate these issues (Au et al., 2013). This type of dataset relying on long cDNA sequences should benefit from our cliff algorithm.

In summary, the work reported here presents an important molecular resource for the community of ascidian developmental biologists as well as algorithms useful to other communities wanting to generate similar resources. These algorithms should be adaptable to the analysis of upcoming third generation sequencing datasets.

**Table:** 

*Terminology*
The following terms used in this study are explained explicitly to avoid possible confusion with similar terms used elsewhere.
*EST:*Expressed Sequence Tag: single pass Sanger sequence from either end of a cloned mRNA.
*EST cluster:* computationally organised and assembled discrete group of ESTs, ideally containing all the ESTs from one gene and no ESTs from other genes.
*Cluster consensus sequence:* predominant sequence determined over the multiple aligned sequences in an EST cluster. Compensates for errors in single pass sequencing and may yield an accurate mRNA sequence.
*Sub-cluster:* an EST cluster may be composed of one layer of sub-clusters. These arise either by joining primary clusters after initial cluster assembly based on paired end data or similarity metrics, or by post-assembly decomposition into distinct transcripts. Sub-clusters have their own consensus sequence, and post-assembly sub-clusters undergo independent ORF analysis.
*Gene model:* physical map of exons and introns identified as belonging to a gene locus. They may be generated by gene modelling computer programs (usual) or manually.
*Transcript model:* gene model for a specific transcript of a gene locus.
*Singleton:* EST sequence not assembled with other EST into a cluster.
*Full-ORF clone:* cDNA clone determined (usually computationally) to contain both the initiator methionine of the encoded protein and the stop codon.
*Gene coverage:* Coverage is a measure of the proportion of gene loci for which we have one or more full-ORF cDNA clones in our GATEWAY collection.

### Funding

This work was supported by the U.S. Department of Energy Joint Genome Institute Office of Science [Contract no. DE-AC02-05CH11231, Community Sequencing Programme 05-SE-04, to P.L]; the United Kingdom Medical Research Council programme [No. A252-5RG70 to M.G.], the French Centre National de la Recherche Scientifique [to P.L., U.R.] and a grant from the Agence Nationale pour la Recherche (TED, ANR-13-BSV2-0011-01).

### Authors' contributions

U.R. and P.L. initiated and guided the study, U.R., P.L. J.M., K.H. and Y.S. provided material; U.R. performed wet lab experiments; M.G., D.S., P.K., F.D., B.L., I.P. and U.R. performed bioinformatics experiments; U.R., M.G., D.S. and P.L. analysed data; U.R., M.G. and P.L. wrote the paper.

## Figures and Tables

**Table 1:** Numbers of clones and clusters affected by novel solutions to the pipeline. These numbers relate to the total of 19,107 clones selected from 26,186 gene clusters and 9380 singletons covering 9083 KH2012 protein coding genes.

**Novel solution**	**Step affected**	**Entity affected**	**Numbers**	**Comments**
Opposite strand splitting	Gene clustering	Cluster	+476	Each split cluster may provide full-ORF clones
Cliff score	5′ end detection	Cluster	+3687	May not be the only evidence used to assess that cluster is full-ORF
SL trans-splicing	5′ end detection	Clone	+5049	All SL read containing cluster are considered to have the 5′ end fo the ORF, irrespective of cliff score
Alternative transcripts	Non-redundant clone selection	Clone	+449	Additional clones selected in case of alternative transcripts
Exon mapping analysis	Final clone list	Clone	−5000	Excessive number of clones mapping to same locus with same exon structure
Manual addition of clones	Final clone list	Clone	+37	Clones for low abundanec developmental genes with known ORF

**Table 2:** Over- and under-represented GO Slim (v1.2, 2008) terms in the KH2010 gene loci associated with one or more full-ORF clones, with corrected *p*-Values<0.01 (see Section 2). Comparison uses only gene loci with associated GO terms: *n*=number of genes in the whole comparison set with this GO term, and *x*=the number of covered genes with the same GO term.

**Covered loci (6645/8188) with GO terms**				
**Over-represented**				
**GO-ID**	**Corr *p*-Value**	***x***	***n***	**Description**
5622	5.35E−17	1183	1323	Intracellular
5737	2.55E−16	536	574	Cytoplasm
8152	1.72E−09	2471	2911	Metabolic process
43,226	1.72E−09	722	810	Organelle
166	5.45E−08	1044	1199	Nucleotide binding
9058	1.79E−07	611	688	Biosynthetic process
6139	1.66E−06	514	578	Nucleobase, nucleoside, nucleotide and nucleic acid metabolic process
44,238	6.06E−06	1786	2108	Primary metabolic process
5634	1.62E−05	315	349	Nucleus
6412	1.81E−05	205	222	Translation
15031	1.16E−04	177	192	Protein transport
3824	2.34E−04	2680	3216	Catalytic activity
5783	3.57E−04	48	48	Endoplasmic reticulum
6350	5.90E−04	57	58	Transcription
3723	1.68E−03	141	154	RNA binding
5654	2.92E−03	37	37	Nucleoplasm
5840	3.14E−03	135	148	Ribosome
8135	6.01E−03	33	33	Translation factor activity, nucleic acid binding
16,043	6.82E−03	152	169	Cellular component organisation
**Under-represented**				
4872	4.85E−26	159	296	Receptor activity
4871	1.83E−18	209	346	Signal transducer activity
30,246	2.30E−11	105	180	Carbohydrate binding
5216	2.30E−11	55	108	Ion channel activity
5509	2.79E−07	270	388	Calcium ion binding
5576	4.96E−04	115	169	Extracellular region
3700	5.73E−04	167	237	Transcription factor activity
5215	6.52E−04	378	509	Transporter activity
30,528	8.03E−04	171	241	Transcription regulator activity
6811	2.27E−03	176	245	Ion transport
5578	3.35E−03	11	23	Proteinaceous extracellular matrix
3774	5.55E−03	42	66	Motor activity

## Supplementary material

Supplementary Fig. 1 Examples of erroneous ORF predictions in KH gene models revealed by full-ORF clone data. The figure presents for three gene loci a comparison of the positions of the ends of the open reading frames in the full-ORF clones and in the KH2012 gene models. In KH gene models, CDS are represented in orange or dark red and UTRs in grey. Picked clone EST sequences are in red. EST cluster consensus sequence are in dark grey and extracted ORF in dark green. Gaps in the assembly (Ns) are in black. (A) The open reading frame of the gene model KH.C6.194 (*cytochrome b-245, beta*) appears to be truncated. The top panel shows a comparison of the cDNA sequence (Query) with the genome sequence (subject) revealing that the genome sequence is missing a single base in what would have been a coding exon. The resulting frame shift is the cause of the truncation. The bottom panel shows that the assembly is erroneous as it abutted two genomic shotgun sequences that should have been separated by a single base. (B) The 5′ boundary of exon 2 of the gene model KH.C6.296 (*dihydrofolate reductase*) appears to be displaced by seven nucleotides, causing a frameshift in exon 1, and consequent mis-translation and truncation of the N-terminus of the model protein. There is no obvious cause in the genome sequence, and it seems likely that the gene modelling program has selected a more canonical, but incorrect, splice acceptor site (C) Two problems with the gene model KH.C1.1191 (*alpha3-fucosyltransferase*): the first 211 nucleotides of the open reading frame are missing, and there are two gene models within the span of the clones. There is a gap in the genome assembly just upstream of the gene, and this is likely where the first exon(s) of the gene would have been found. The gene model KH.C1.1191 thus yields an N-terminal truncation of 71 residues. The gene model KH.C1.626 lies entirely within the mature transcripts in the 3′ UTR of this gene, and that the apparent open reading frame of 243 nucleotides is a chance occurrence of a long stretch of DNA, as it matches no know protein in any species by Blastp analysis, and even shows no match to the closely related *Ciona savignyi* genome by low stringency (*e*-05) tBlastn alignment.

Supplementary Fig. 2 The cliff algorithm identifies full-ORF *Xenopus tropicalis* models. The figure shows the distribution and relative position of the cliff peak and the initiator methionine in *Xenopus tropicalis* (A) and *Ciona intestinalis* (B). The horizontal axis indicates the distance from cliff to initiator ATG (10 nt bins). Positive values correspond to a cliff positioned 5′ to the ATG as expected, negative values correspond to an ATG positioned 5′ to the cliff, suggesting premature stop of the Reverse transcriptase, perhaps due to RNA secondary structures. The vertical axis gives numbers of full-ORF EST clusters at each distance. The upper panel (in each pair) corresponds to clusters in which the ORF is preceded by in frame stop codons. The bottom panel corresponds to ORFs without in frame upstream stop codon. The analyses in *Xenopus* were done using ~1,220,000 ESTs and 6777 full-ORF clusters. The average 5′ UTR length without in frame ORFs is 43 bp in *Xenopus* (39 bp in *Ciona*), while the average 5′ UTR length with in frame Stop is 138 bp in *Xenopus* (72 bp in *Ciona*).
