# Supplementary material for: A pipeline for the systematic identification of non-redundant full-ORF cDNAs for polymorphic and evolutionary divergent genomes: Application to the ascidian Ciona intestinalis
Source: Dev Biol. 2015 Aug 15;404(2):149–63. doi: 10.1016/j.ydbio.2015.05.014 (PMC4528069; doi:10.1016/j.ydbio.2015.05.014)
Supplement: Supplementary file 1 — Supplementary Fig. 1 Examples of erroneous ORF predictions in KH gene models revealed by full-ORF clone data. The figure presents for three gene loci a comparison of the positions of the ends of the open reading frames in the full-ORF clones and in the KH2012 gene models. In KH gene models, CDS are represented in orange or dark red and UTRs in grey. Picked clone EST sequences are in red. EST cluster consensus sequence are in dark grey and extracted ORF in dark green. Gaps in the assembly (Ns) are in black. (A) The open reading frame of the gene model KH.C6.194 (cytochrome b-245, beta) appears to be truncated. The top panel shows a comparison of the cDNA sequence (Query) with the genome sequence (subject) revealing that the genome sequence is missing a single base in what would have been a coding exon. The resulting frame shift is the cause of the truncation. The bottom panel shows that the assembly is erroneous as it abutted two genomic shotgun sequences that should have been separated by a single base. (B) The 5′ boundary of exon 2 of the gene model KH.C6.296 (dihydrofolate reductase) appears to be displaced by seven nucleotides, causing a frameshift in exon 1, and consequent mis-translation and truncation of the N-terminus of the model protein. There is no obvious cause in the genome sequence, and it seems likely that the gene modelling program has selected a more canonical, but incorrect, splice acceptor site (C) Two problems with the gene model KH.C1.1191 (alpha3-fucosyltransferase): the first 211 nucleotides of the open reading frame are missing, and there are two gene models within the span of the clones. There is a gap in the genome assembly just upstream of the gene, and this is likely where the first exon(s) of the gene would have been found. The gene model KH.C1.1191 thus yields an N-terminal truncation of 71 residues. The gene model KH.C1.626 lies entirely within the mature transcripts in the 3′ UTR of this gene, and that the apparent open reading frame o [file mmc1.pptx]

## Slide 1
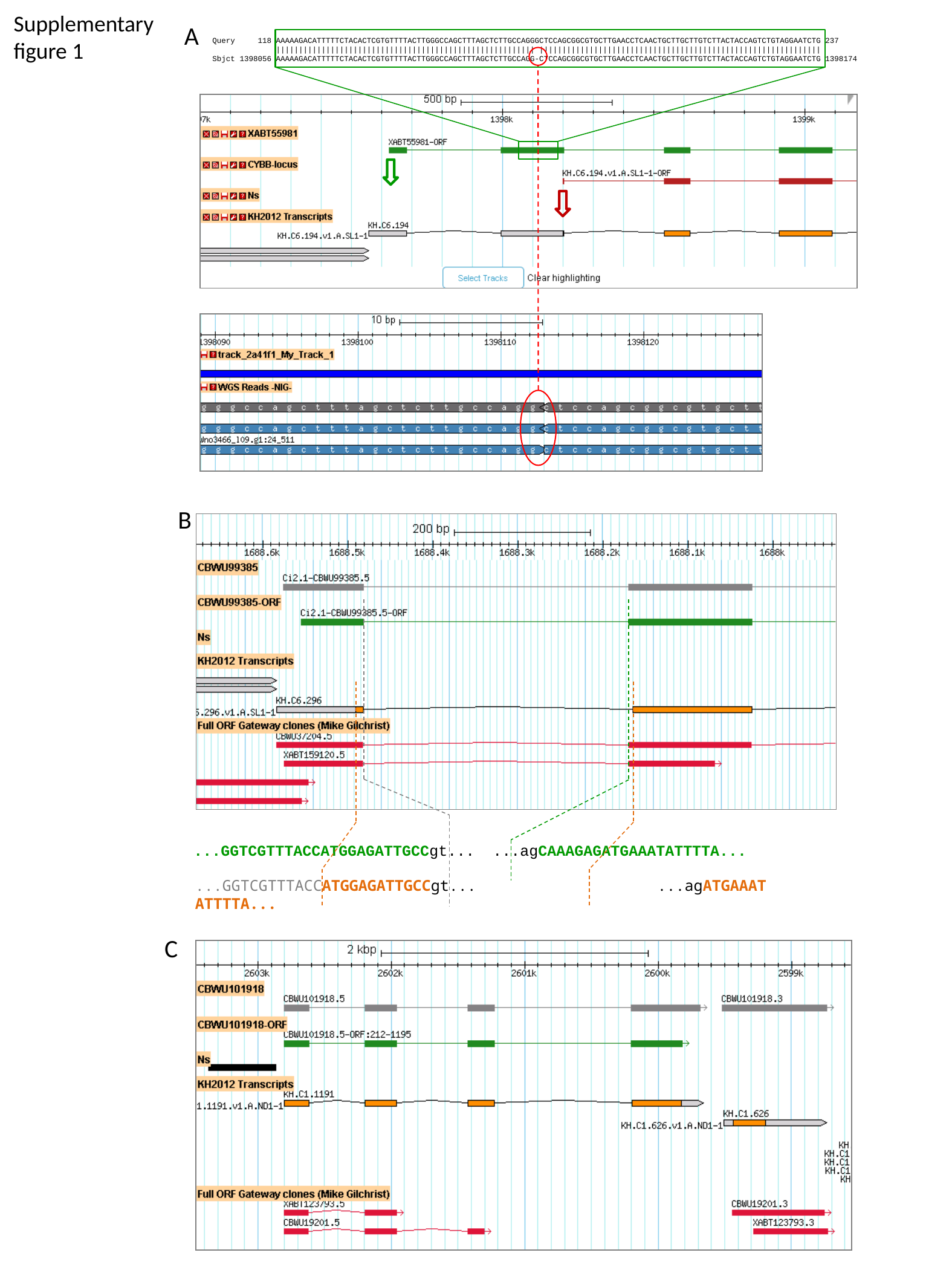

Supplementary
figure 1
A
Query 118 AAAAAGACATTTTTCTACACTCGTGTTTTACTTGGGCCAGCTTTAGCTCTTGCCAGGGCTCCAGCGGCGTGCTTGAACCTCAACTGCTTGCTTGTCTTACTACCAGTCTGTAGGAATCTG 237
 ||||||||||||||||||||||||||||||||||||||||||||||||||||||||| ||||||||||||||||||||||||||||||||||||||||||||||||||||||||||||||
Sbjct 1398056 AAAAAGACATTTTTCTACACTCGTGTTTTACTTGGGCCAGCTTTAGCTCTTGCCAGG-CTCCAGCGGCGTGCTTGAACCTCAACTGCTTGCTTGTCTTACTACCAGTCTGTAGGAATCTG 1398174
B
...GGTCGTTTACCATGGAGATTGCCgt... ...agCAAAGAGATGAAATATTTTA...
...GGTCGTTTACCATGGAGATTGCCgt... ...agATGAAATATTTTA...
C
