# Supplementary material for: A pipeline for the systematic identification of non-redundant full-ORF cDNAs for polymorphic and evolutionary divergent genomes: Application to the ascidian Ciona intestinalis
Source: Dev Biol. 2015 Aug 15;404(2):149–63. doi: 10.1016/j.ydbio.2015.05.014 (PMC4528069; doi:10.1016/j.ydbio.2015.05.014)
Supplement: Supplementary file 2 — Supplementary Fig. 2 The cliff algorithm identifies full-ORF Xenopus tropicalis models. The figure shows the distribution and relative position of the cliff peak and the initiator methionine in Xenopus tropicalis (A) and Ciona intestinalis (B). The horizontal axis indicates the distance from cliff to initiator ATG (10 nt bins). Positive values correspond to a cliff positioned 5′ to the ATG as expected, negative values correspond to an ATG positioned 5′ to the cliff, suggesting premature stop of the Reverse transcriptase, perhaps due to RNA secondary structures. The vertical axis gives numbers of full-ORF EST clusters at each distance. The upper panel (in each pair) corresponds to clusters in which the ORF is preceded by in frame stop codons. The bottom panel corresponds to ORFs without in frame upstream stop codon. The analyses in Xenopus were done using ~1,220,000 ESTs and 6777 full-ORF clusters. The average 5′ UTR length without in frame ORFs is 43 bp in Xenopus (39 bp in Ciona), while the average 5′ UTR length with in frame Stop is 138 bp in Xenopus (72 bp in Ciona). [file mmc2.pptx]

## Slide 1
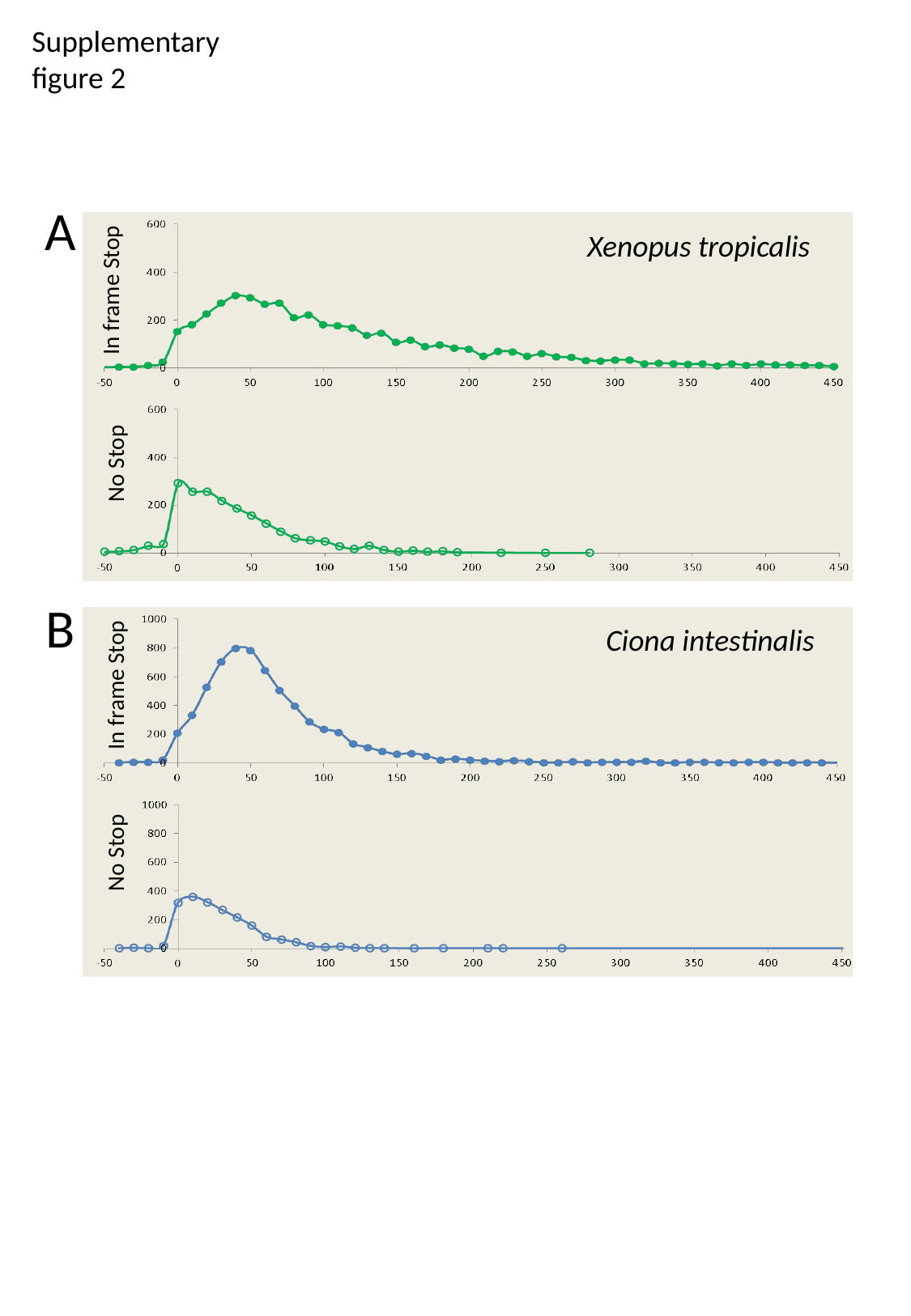

Supplementary
figure 2
A
Xenopus tropicalis
In frame Stop
No Stop
B
Ciona intestinalis
In frame Stop
No Stop
